# Medium- and Long-Term Evaluation of Splenic Arterial Embolization: A Retrospective CT Volumetric and Hematologic Function Analysis

**DOI:** 10.3390/jpm15090424

**Published:** 2025-09-04

**Authors:** Filippo Piacentino, Federico Fontana, Cecilia Beltramini, Andrea Coppola, Anna Maria Ierardi, Gianpaolo Carrafiello, Giulio Carcano, Massimo Venturini

**Affiliations:** 1 Department of Diagnostic and Interventional Radiology, Circolo Hospital and Macchi Foundation, Insubria University, 21100 Varese, Italy; federico.fontana@uninsubria.it (F.F.);; 2 Department of Medicine and Technological Innovation (DiMIT), Insubria University, 21100 Varese, Italy; 3 Interventional Radiology Unit, Department of Radiology, Foundation IRCCS Ca’ Granda-Ospedale Maggiore Policlinico, 20126 Milan, Italy; annamaria.ierardi@policlinico.mi.it (A.M.I.);; 4 General Emergency and Transplant Surgery Unit, ASST Settelaghi, 21100 Varese, Italy

**Keywords:** splenic arterial embolization, CT volumetry, trauma, aneurysm, interventional radiology

## Abstract

**Background:** Splenic arterial embolization (SAE) is a well-established technique in the non-operative management of splenic trauma and aneurysms. While its short-term safety and efficacy have been widely documented, medium- and long-term impacts on splenic volume and function remain under-investigated. This study aimed to evaluate volumetric changes and hematological parameters following SAE, with emphasis on its role in preserving splenic integrity and potential integration with AI-enhanced imaging technologies. **Methods:** We retrospectively analyzed 17 patients treated with SAE between January 2014 and December 2023. Volumetric measurements were performed using computed tomography (CT) with 3D reconstructions before and after SAE. Patients were divided into two groups based on indication: polytrauma (n = 8) and splenic artery aneurysm (n = 9). Hematological parameters including white blood cells (WBCs), red blood cells (RBCs), and hemoglobin (Hb) were evaluated in correlation with clinical outcomes. Statistical significance was assessed using Student’s *t*-test, and power analysis was conducted. **Results:** Among the trauma group, mean splenic volume decreased from 190.5 ± 51.2 cm^3^ to 147.8 ± 77.8 cm^3^ (*p* = 0.2158), while in the aneurysm group, volume decreased from 195.4 ± 78.9 cm^3^ to 143.7 ± 81.4 cm^3^ (*p* = 0.184). Though not statistically significant, these changes suggest post-procedural splenic remodeling. The technical success of SAE was 100%, with no cases of late follow-up infarction, abscess, immunological impairment, or secondary splenectomy required. Hematologic parameters remained within normal limits in follow-up assessments. **Conclusions:** SAE represents a safe and effective intervention for spleen preservation in both traumatic and aneurysmal conditions. Although a reduction in splenic volume has been observed, white blood cell counts, a reliable indicator of splenic function, have remained stable over time. This finding supports the preservation of splenic function following SAE.

## 1. Introduction

The spleen is a highly vascularized abdominal organ essential to hematologic and immunologic function. It plays a central role in filtering aged red blood cells (RBCs), mounting immune responses to blood-borne pathogens, and serving as a reservoir for various immune cells. Due to its fragile parenchyma and rich blood supply, the spleen is particularly susceptible to injury following blunt abdominal trauma [1,2]. In such cases, management has evolved significantly over recent decades—from routine splenectomy to a more conservative, organ-preserving approach [3,4,5,6].

Splenic artery embolization (SAE) has become a widely accepted non-operative treatment for hemodynamically stable patients with high-grade splenic injuries, as well as a prophylactic or therapeutic approach for splenic artery aneurysms (SAAs). SAE offers the advantage of targeted vascular occlusion, minimizing parenchymal damage while preserving the immunological function of the spleen. Numerous studies have documented SAE’s effectiveness in controlling hemorrhage and reducing the need for surgical intervention, using a wide array of embolic agents [7,8,9,10,11,12,13]. However, concerns remain regarding potential complications such as splenic infarction, abscess, and delayed rupture. Additionally, long-term impacts on splenic architecture and hematologic function are not fully understood.

In recent years, computed tomography (CT)-based volumetry has emerged as a reliable technique for evaluating organ size and structural changes over time [14,15]. When coupled with semi-automated or AI-enhanced segmentation, CT volumetry enables objective quantification of splenic changes after embolization. This is particularly relevant in assessing functional outcomes, as a reduction in organ volume may suggest tissue infarction or remodeling. Nevertheless, few studies have systematically assessed splenic volume alongside hematologic parameters in the months following SAE, despite the rationale that hematologic markers—particularly white blood cell counts—serve as established indicators of splenic function. Evaluating splenic volume in isolation may be of limited significance, as the preservation of splenic size without a corresponding maintenance of functional capacity would be clinically inconsequential.

Furthermore, with the advent of artificial intelligence (AI) in medical imaging, there is growing potential to apply machine learning algorithms to predict post-embolization outcomes, assist in volumetric analysis, and personalize follow-up protocols [16,17,18,19,20]. Recent evidence further underscores that AI-driven image analysis has the potential not only to improve diagnostic accuracy and reproducibility, but also to provide prognostic insights that may guide interventional strategies. In particular, AI-enhanced volumetric assessment can support the early detection of parenchymal alterations, predict treatment-related outcomes, and facilitate tailored follow-up protocols, thereby offering clinicians robust quantitative tools for personalized patient management in splenic interventions [21].

The aim of this study is to evaluate medium- and long-term splenic changes in patients who underwent SAE for either trauma or aneurysmal disease. By analyzing volumetric data and hematologic profiles, we aim to assess the impact of SAE on splenic integrity and identify potential applications of AI-based imaging tools in future clinical practice.

## 2. Materials and Methods

### 2.1. Study Design

We performed a retrospective, observational study analyzing the medical records and radiological exams of 50 patients who underwent SAE between January 2014 and December 2023. From this initial cohort, 17 patients were selected based on specific inclusion and exclusion criteria as shown in Table 1. 

### 2.2. Inclusion and Exclusion Criteria

Inclusion criteria were confirmed indication for SAE due to either traumatic splenic injury or splenic artery aneurysm, availability of pre- and post-procedural CT scans suitable for volumetric analysis, and availability of hematological data pre- and post-embolization. Exclusion criteria included lack of imaging and hematological follow-up.

### 2.3. SAE Procedure

All embolization procedures were performed by interventional radiologists using a femoral approach and digital subtraction angiography. Embolic agents included coils (Azur, Terumo Corp., Tokyo, Japan) (Penumbra Inc., Alameda, CA, USA), non-adhesive liquid embolic agents (Onyx, ev3 Endovascular, Inc. Plymouth, MN, USA) and temporary embolic agents (Spongostan sponge, Johnson & Johnson Medical, New Brunswick, NJ, USA). Embolization strategies were selected based on vascular anatomy, hemodynamic stability, and the location of the injury or aneurysm. In the trauma group, six patients underwent distal branch embolization, while two received main artery coiling. In the SAA group, six patients were treated with endovascular ligation using the front door–back door technique, and in three cases, embolization was performed using a packing technique to exclude only the aneurysm’s sac.

The choice of embolic material was further guided by the type of lesion being treated. In particular, for pseudoaneurysms, given their narrow-neck morphology in the cases addressed, coils were preferred in order to achieve exclusion of the aneurysmal sac. In instances of active bleeding detected during angiography, non-adhesive liquid embolic agents, coils, or a combination of both were used, depending on the severity of hemorrhage and the expertise of the interventional radiologist, with the aim of reducing bleeding as visualized on digital subtraction angiography [22,23].

### 2.4. Imaging and Volumetric Analysis

Volumetric data were obtained from the hospital PACS system (Agfa HealthCare, Cinisello Balsamo (MI), Milan, Italy). Using 3D reconstruction software (IntelliSpace v. 12.1, Philips HealthCare. Best, The Netherlands), the spleen was segmented manually, excluding devascularized areas (e.g., infarcts or lacerations) in the pre-procedural contrast-enhanced CT. For the post-procedural assessment, spleen volume was manually calculated using the same software, considering any CT scan that included the spleen, regardless of whether contrast was administered, performed in relation to subsequent health issues. Volume was measured in cubic centimeters (cm^3^). Measurements were performed by an interventional radiologist with ≥15 years of experience.

### 2.5. Hematological Data

Laboratory values (WBC, RBC, Hb) were extracted from the clinical database and compared pre- and post-SAE at 1 month, 3 months, and 6 months.

### 2.6. Statistical Analysis

Statistical analysis was conducted using IBM SPSS Statistics Software version 28.0 (Chicago, IL, USA). Data are reported as mean ± standard deviation for normally distributed variables and as median ± interquartile range for non-normally distributed variables. Normality was assessed using the Shapiro–Wilk test. For variables that followed a normal distribution, Student’s *t*-test was applied to compare volumetric and hematologic differences. For non-normally distributed data—such as WBC counts in both the polytrauma and SAA groups—the Mann–Whitney U test was used. A *p*-value < 0.05 was considered statistically significant. Power analysis was performed using GPower 3.1* to ensure adequate sample size and statistical reliability. In order to analyze splenic volumetry and white blood cell counts before and after embolization according to the embolization technique used, statistical analysis was conducted: data are reported as mean ± standard deviation for normally distributed variables and as median ± interquartile range for non-normally distributed variables. Normality of distribution was assessed using the Shapiro–Wilk test. For variables that followed a normal distribution, a paired Student’s *t*-test was applied to compare pre- and post-embolization volumetric and hematologic data. For non-normally distributed variables, the Wilcoxon signed-rank test was used instead. A *p*-value < 0.05 was considered statistically significant.

## 3. Results

### 3.1. Embolization Technique and Agents Used

In the polytrauma group, five distal (62.5%) and three proximal (37.5%) embolizations were performed. In four patients only coils were used; in three patients, Onyx alone was used; and in one patient, a combination of coils and Onyx 34 was employed.

In the group with SAAs, three patients underwent SAE with coils alone, whereas in the remaining six patients, a combination of coils and Onyx 34 was used.

### 3.2. CT Volumetry

Based on the dates of the CT scans performed for each patient, the average time interval between the pre-procedural and post-procedural imaging was 1711 days.

In the group of polytrauma patients, the variable volume showed a normal distribution according to the Shapiro–Wilk test both before and after the embolization procedure, with *p*-values of 0.633 and 0.918, respectively. The volume measured 190.5 ± 51.2 cm^3^ (mean ± SD) before embolization and 147.8 ± 77.8 cm^3^ after. Student’s *t*-test yielded a *p*-value of 0.2158, with a priori power of 0.154. Therefore, no statistically significant differences were observed, as shown in Table 2.

In the group of patients with SAAs, the volume variable was normally distributed both before and after embolization (Shapiro–Wilk test *p*-values of 0.771 and 0.242, respectively). The volume measured 195.4 ± 78.9 cm^3^ before embolization and 143.7 ± 81.4 cm^3^ after. Student’s *t*-test produced a *p*-value of 0.184, with a priori power of 0.167. Thus, no statistically significant differences were detected, as shown in Table 2 (Table 2).

**Figure 1:**
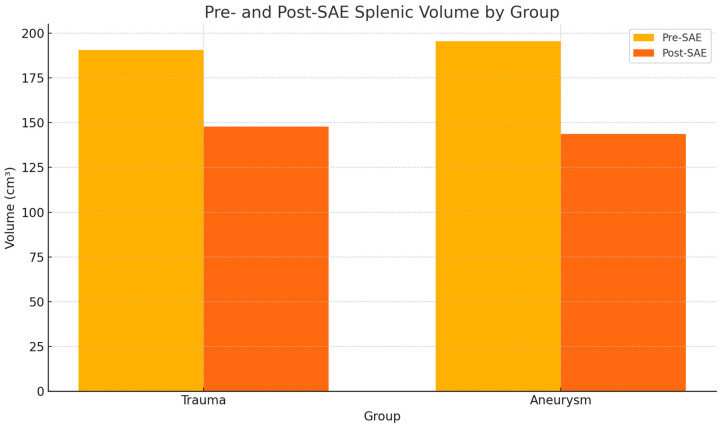
Pre- and post-splenic artery embolization (SAE) spleen volume by group. The volume did not change significantly within either group before and after embolization.

**Figure 2:**
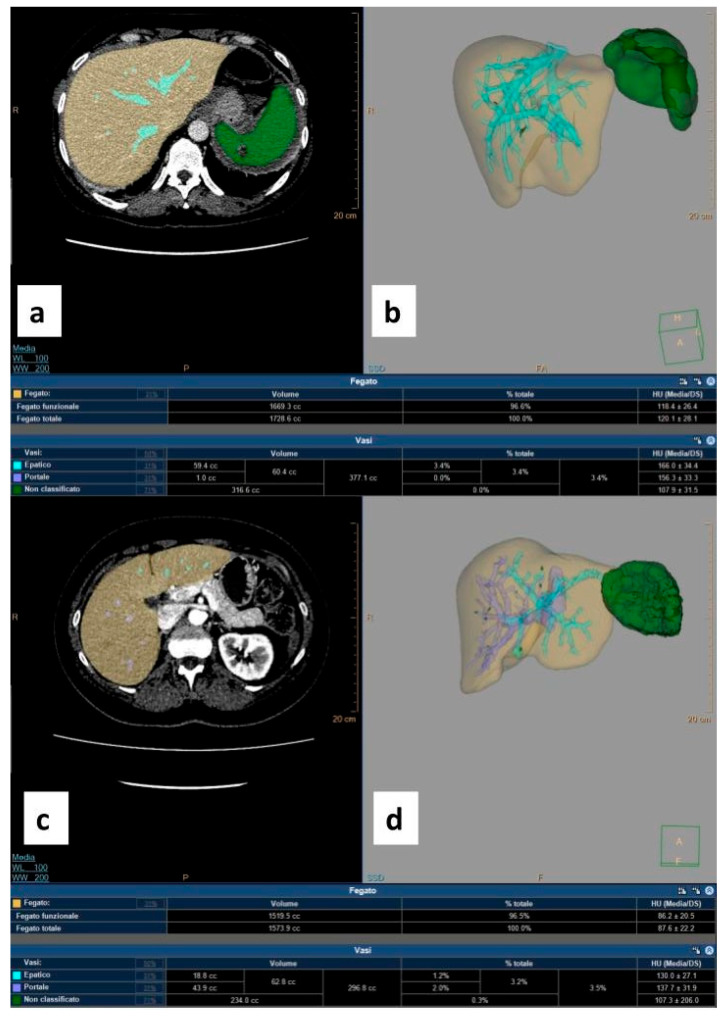
Three-dimensional reconstruction CT-based images obtained using IntelliSpace software v. 12.1. (**a**) Pre-procedural contrast-enhanced axial CT scan acquired during the venous phase shows the hepatic parenchyma in yellow and the healthy splenic parenchyma in green; lacerated–contused areas are not colored. (**b**) Pre-procedural volumetric rendering of the splenic parenchyma in green with the corresponding result in the table (healthy splenic volume: 316.6 cc). (**c**) Post-splenic artery embolization (SAE) contrast-enhanced axial CT scan acquired during the venous phase shows the hepatic parenchyma in yellow and the residual healthy splenic parenchyma in green. (**d**) Post-SAE volumetric rendering of the splenic parenchyma in green with the corresponding result in the table (residual splenic volume: 234 cc).

Figure 1 shows a bar graph comparing pre- and post-SAE spleen volumes by group (Figure 1) and Figure 2 shows an example of a CT-based 3D reconstruction that was performed for each patient (Figure 2).

Table 3 shows the statistical comparison of splenic volume before and after treatment conducted across three patient groups, categorized according to the embolization technique used.

In the NALEA group, splenic volumes before and after embolization were normally distributed; median values were 219.6 ± 62.3 cm^3^ before embolization and 163.2 ± 84.5 cm^3^ after. The paired Student’s *t*-test yielded a *p*-value < 0.05, indicating a statistically significant reduction in splenic volume, as shown in Table 3.

In the coil-only group, splenic volumes were non-normally distributed; median values were 205.3 ± 54.1cm^3^ before and 161.2 ± 76.1 cm^3^ after embolization. The Wilcoxon test returned a *p*-value < 0.05, confirming a statistically significant but more modest reduction, as shown in Table 3.

In the combination group, splenic volumes were normally distributed; median values were 218.3 ± 59.2 before and 163.8 ± 77.4cm^3^ after. The paired Student’s *t*-test yielded a *p*-value < 0.05, indicating a significant intermediate reduction in volume, as shown in Table 3.

### 3.3. Hemocytometric Analysis

#### 3.3.1. White Blood Cells

In the polytrauma group, WBC counts before and after embolization were not normally distributed (Shapiro–Wilk *p*-values of 0.005 and <0.001, respectively). Median values were 17.5 ± 5.2 × 10^3^ U/mm^3^ before embolization and 12.5 ± 3.4 × 10^3^ U/mm^3^ after. The Mann–Whitney U test yielded a *p*-value of 0.777. Hence, no statistically significant difference was observed, as shown in Table 4.

In the group with SAAs, WBC counts were also non-normally distributed (*p* = 0.039 and <0.001 before and after embolization, respectively). The median values were 9.0 ± 3.9 × 10^3^ U/mm^3^ before and 9.3 ± 6.7 × 10^3^ U/mm^3^ after embolization. The Mann–Whitney U test returned a *p*-value of 0.285, indicating no statistically significant difference, as shown in Table 4 (Table 4).

**Figure 3:**
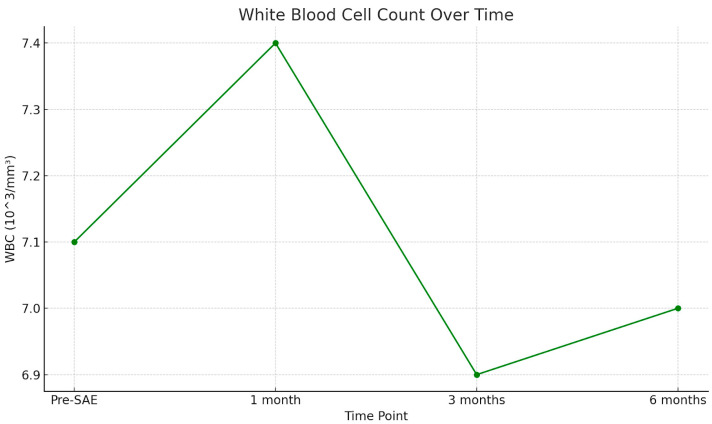
White blood cell (WBC) counts are measured at four time points: pre-SAE, 1 month, 3 months, and 6 months post-procedure. The WBC count remains stable over time, suggesting no significant immunological impairment following splenic arterial embolization.

Figure 3 shows a scatter plot illustrating the trend of white blood cell (WBC) count over time (Figure 3).

Table 5 shows the statistical comparison of WBC count before and after treatment across three patient groups, categorized according to the embolization technique used.

In the NALEA group, WBC counts before and after embolization were non-normally distributed; median values were 10.1 ± 5.6 × 10^9^/L before embolization and 8.9 ± 2.6 × 10^9^/L after. The Wilcoxon test yielded a *p*-value < 0.05, indicating a statistically significant increase in WBC count, as shown in Table 5.

In the coil-only group, WBC counts were normally distributed; median values were 9.4 ± 5.0 × 10^9^/L before and 8.8 ± 2.4 × 10^9^/L after embolization. The paired Student’s *t*-test returned a *p*-value > 0.05, showing a slight increase, non-statistically significant, as shown in Table 5.

In the combination group, WBC counts were non-normally distributed; median values were 9.9 ± 5.4 × 10^9^/L before and 8.9 ± 2.5 × 10^9^/L after. The Wilcoxon test yielded a *p*-value < 0.05, indicating a significant moderate increase in WBC count, as shown in Table 5.

#### 3.3.2. Red Blood Cells

In the polytrauma group, RBC counts were not normally distributed before or after embolization (Shapiro–Wilk *p*-values < 0.001 for both). Median values were 4.5 ± 1.4 × 10^6^ U/mm^3^ before and 6.0 ± 0.6 × 10^6^ U/mm^3^ after embolization. The Mann–Whitney U test returned a *p*-value of 0.835, indicating no statistically significant difference.

In the group with SAAs, RBC counts were also not normally distributed (Shapiro–Wilk *p*-values < 0.001 and 0.001, respectively). Median values were 8.5 ± 1.1 × 10^6^ U/mm^3^ before and 11.0 ± 0.4 × 10^6^ U/mm^3^ after embolization. The Mann–Whitney U test yielded a *p*-value of 0.987, showing no significant differences.

#### 3.3.3. Hemoglobin

In the polytrauma group, Hb values were not normally distributed (Shapiro–Wilk *p* = 0.002 before, and <0.001 after embolization). Median hemoglobin levels were 14.0 ± 2.6 g/dL before and 10.5 ± 1.2 g/dL after embolization. The Mann–Whitney U test gave a *p*-value of 0.124, indicating no significant difference.

In the SAAs, Hb levels were also not normally distributed (*p* < 0.001 before and *p* = 0.043 after embolization). Median values were 14.0 ± 2.5 g/dL before and 10.0 ± 2.2 g/dL after embolization. The Mann–Whitney U test revealed a statistically significant difference, with a *p*-value of <0.001.

### 3.4. Technical and Clinical Success

Technical success, defined as correct and effective execution of the SAE procedure according to established protocols, was achieved in 100% of cases. The complication rate in the studied population was 35.3%; specifically, five patients developed partial splenic ischemia post-procedure (pharmacologically treated with regard to pain and risk of abscess formation, with complete resolution in all cases during long-term follow-up), and one patient developed a splenic abscess (treated with percutaneous drainage and antibiotic therapy, with complete resolution at 13 days).

Clinical success, defined as patient survival following the traumatic event due to the endovascular procedure, was also 100%. No patient deaths occurred as a result of the procedure or during the medium- to long-term follow-up period.

**Figure 4:**
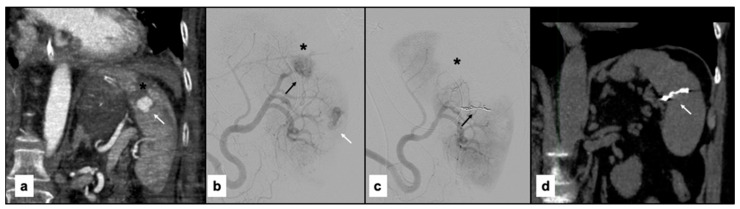
(**a**) Contrast-enhanced paracoronal CT scan acquired during the arterial phase shows a large laceration of the superior splenic pole (black asterisk) associated with a sizable pseudoaneurysm (arrow). (**b**) Digital subtraction angiography (DSA) performed using a 5F Cobra-shaped catheter (Cordis, FL, USA) confirms the laceration of the superior splenic pole (black asterisk) and the presence of the pseudoaneurysm (black arrow) previously identified on CT, with heterogeneous intraparenchymal vascularization (white arrow). (**c**) Post-procedural DSA performed through a guiding catheter demonstrates embolization of a branch feeding the vascular lesion using Penumbra coils (Crossmed, Turin, Italy) (black arrow), resulting in marked devascularization of the splenic parenchyma (black asterisk). (**d**) Follow-up non-contrast paracoronal CT reconstruction shows partial recovery of splenic volume in the area treated with the coils (white arrow).

**Figure 5:**
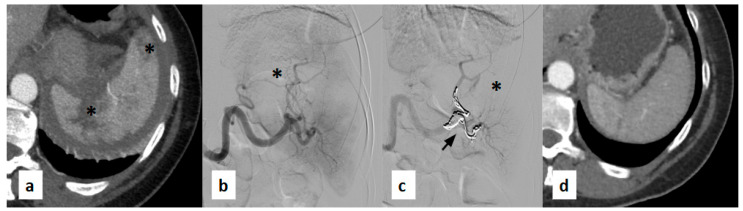
(**a**) Contrast-enhanced axial CT scan acquired during the arterial phase shows lacerated-contused areas with extensive full-thickness multilinear laceration extending from the hilum (black asterisks). (**b**) Digital subtraction angiography (DSA) performed using a 5F Cobra-shaped catheter (Cordis, FL, USA) confirms the full-thickness hilar laceration of the spleen (black asterisk) and altered intraparenchymal vascularization previously identified on CT. (**c**) Post-procedural DSA performed through a guiding catheter demonstrates embolization of the branches feeding the hypervascular areas using Penumbra coils (Crossmed, Turin, Italy) (black arrow), resulting in marked reduction of vascularization of the splenic parenchyma (black asterisk). (**d**) Follow-up contrast CT scan reconstruction shows partial recovery of splenic volume with an almost homogeneous contrast enhancement of the residual splenic parenchyma.

Figure 4 and Figure 5 show examples of the diagnostic phase, treatment with splenic artery embolization, and follow-up imaging performed for the patients included in the study (Figure 4 and Figure 5).

### 3.5. Length of Hospital Stay

In the polytrauma group, the average hospital stay was 18.1 ± 9.8 days. In the SAA group, the average hospital stay was 4.7 ± 3.4 days.

## 4. Discussion

This study provides valuable insights into the medium- and long-term outcomes of splenic arterial embolization (SAE) in patients with blunt abdominal trauma and splenic artery aneurysms. Our findings suggest that SAE, performed with either a proximal or distal technique using various embolic agents, effectively preserves splenic volume and hematologic function over time. The minor, non-significant reduction in splenic volume across both patient groups is consistent with the previous literature suggesting remodeling rather than infarction or functional loss [24,25,26]. The recovery of splenic volume following arterial embolization procedures has been documented in the literature, albeit with notable distinctions. Specifically, it appears that only patients who underwent proximal splenic artery embolization (SAE) demonstrated progressive volume recovery over time [24,25,26]. This phenomenon is likely attributable to collateral blood supply to the spleen, typically via the gastric, gastroepiploic, and omental vessels. In contrast, patients who underwent distal SAE did not exhibit the same degree of recovery. This difference is probably due to the lack of an extensive collateral network distal to the embolized vessel, limiting the spleen’s ability to regenerate its parenchyma.

A retrospective study involving 148 patients reported an average splenic volume reduction of 75.1 cm^3^ (−15.82%) at 8-year follow-up. Another study by Bell et al. found an average decrease of 44.14 cm^3^, with volume reduction observed after distal embolization and an increase following proximal embolization [27,28].

The results of our study show that splenic volume does not change significantly after SAE compared to baseline values. The overall average volume reduction observed during the follow-up period was 43.1 cm^3^ (−22.3%) over a follow-up ranging from 1 to 5 years. In our case series, we did not observe any significant differences in splenic volume or function between patients treated with proximal or distal embolization, suggesting that preserving at least one collateral vascular pathway may be sufficient to ensure organ recovery in the medium to long term. The results of the evaluation of volumetric changes and WBC count, stratified according to the type of embolic agent used, demonstrated a more pronounced effect on both splenic volume reduction and WBC increase in patients treated with non-adhesive liquid embolic agents (NALEA). These findings may primarily be explained by the rapid distribution kinetics of NALEA, which enable an immediate and complete vascular occlusion. Conversely, the group of patients treated with coils showed a more modest impact on both splenic volume and WBC count. This is likely related to the mechanism of action of coils, which provides a slower and less complete occlusion. In addition, in the study population, coils were predominantly employed for the treatment of SAAs, rather than for diffuse parenchymal embolization.

In our opinion, a key strength of this study is the use of CT-based volumetric analysis, which allowed for objective and reproducible assessment of splenic morphology even without CECT. As documented in the literature, the exclusion of infarcted tissue and parenchymal hematomas provides a realistic evaluation of residual functional splenic volume [29].

Furthermore, the absence of statistically significant changes in WBC, RBC, and Hb values over a six-month follow-up supports the hypothesis that SAE does not induce long-term hematologic suppression or immunologic compromise. Notably, despite an observed average reduction of approximately 20% in splenic volume over time, no corresponding decrease in WBC count was detected—an important finding, given that WBC count is widely considered a reliable marker of splenic function. In particular, as shown in Figure 2, WBC levels demonstrated a transient increase at 1 month post-SAE, a period that typically includes hospitalization. This early elevation is most likely attributable to hospital-associated infections, post-traumatic stress response, or reactive bone marrow hyperproliferation. At 3 months, WBC counts declined, suggesting resolution of the acute phase and normalization of the inflammatory or stress-related response. By 6 months, WBC levels appeared to plateau, returning to values comparable to baseline, further supporting the idea of preserved and recovering hematologic homeostasis following SAE.

Importantly, our results support the high technical and clinical success rates of SAE, which align with prior studies reporting success rates of 95–100%. The lack of post-procedural complications such as abscess, infarction, or delayed hemorrhage further strengthens the safety profile of the intervention [8,30].

An emerging area of interest is the application of artificial intelligence (AI) and machine learning in radiologic assessment. AI can potentially enhance organ segmentation and volumetric precision, particularly in complex trauma cases with distorted anatomy [31,32]. AI-based algorithms may also predict complications or guide personalized follow-up schedules based on patient-specific imaging patterns, lab trends, and clinical parameters. In the context of this study, integration of AI tools could automate future volumetric analyses, reduce interobserver variability, and potentially enable real-time risk stratification.

Beyond volumetric analysis, AI applications could also assist in planning the embolization procedure itself. Advanced algorithms may enable pre-procedural segmentation of the splenic parenchyma according to vascular territories, allowing interventional radiologists to better anticipate the extent of parenchymal devascularization for a given embolization site. This could, in turn, facilitate estimation of the degree of post-procedural necrosis and support intra-procedural decision-making. From an organizational perspective, integration of AI-based tools into PACS platforms such as Philips IntelliSpace could streamline image post-processing, reduce reporting time, and standardize volumetric assessment across institutions. Clinically, predictive AI models could combine imaging, hematologic trends, and patient-specific parameters to stratify the risk of complications, guide follow-up frequency, and potentially forecast long-term functional outcomes. Taken together, these developments suggest that AI has the potential to enhance both the technical workflow and the clinical management of patients undergoing SAE, ultimately contributing to more personalized and efficient care pathways.

This study has several limitations. First, the retrospective design inherently limits causal inferences and is prone to selection bias. Second, the sample size (n = 17) is small, reducing statistical power and generalizability. Larger, multicentric studies are needed to confirm our findings and detect potentially significant differences. Third, manual segmentation, while performed by experienced radiologists, is time-consuming and subject to observer variability. Incorporation of automated or semi-automated AI-based segmentation tools may improve consistency in future research. Lastly, we did not include specific immune function assays (e.g., opsonization or vaccine response), which may offer a more precise measure of splenic functional integrity.

Future studies should explore the integration of AI tools into imaging workflows and examine their role in predicting long-term clinical outcomes. Prospective trials evaluating immunologic function post-SAE, including responses to vaccines and incidence of infections, will also be valuable. In addition, longer follow-up (beyond 6–12 months) would help assess the durability of splenic volume and function.

## 5. Conclusions

SAE remains a safe and effective organ-preserving treatment for both traumatic splenic injury and SAAs. It is associated with excellent long-term outcomes, including stable hematologic parameters and preserved immunologic function. Although a modest reduction in splenic volume was observed over time, this did not correspond to a decline in WBC count, a key indicator of splenic function. In fact, WBC levels showed only a transient post-procedural increase followed by normalization over subsequent months, indicating preserved hematologic homeostasis. These findings could support SAE as a possible alternative to splenectomy, particularly in patients for whom preservation of immune function is essential. The future integration of AI into radiologic workflows may further improve procedural precision, support individualized patient care, and broaden the applicability of image-guided embolization techniques in both emergency and elective settings.

## Figures and Tables

**Table 1:** Patient characteristics.

Group	N	Male (%)	Female (%)	Mean Age ± SD (Years)
Trauma	8	62.5	37.5	61.4 ± 11.9
Aneurysm	9	55.5	44.5	67.8 ± 14.2

**Table 2:** Statistical analysis of CT volumetric measurements before and after embolization.

Patient Group	Pre-SAE Volume (cm^3^)	Post-SAE Volume (cm^3^)	Shapiro–Wilk *p*-Value (Pre)	Shapiro–Wilk *p*-Value (Post)	Student’s *t*-Test *p*-Value	A Priori Power
Polytrauma	190.5 ± 51.2	147.8 ± 77.8	0.633	0.918	0.2158	0.154
Splenic Artery Aneurysm	195.4 ± 78.9	143.7 ± 81.4	0.771	0.242	0.184	0.167

**Table 3:** Statistical analysis of CT volumetric measurements before and after embolization by technique.

Embolization Technique	Pre-SAE Volume (cm^3^)	Post-SAE Volume (cm^3^)	Δ Volume	Test	*p*-Value
NALEA	219.6 ± 62.3	163.2 ± 84.5	−56.4 ± 84.3	Student’s *t*-test	0.043
Coils	205.3 ± 54.1	161.2 ± 76.1	−44.1 ± 78.3	Wilcoxon signed-rank	0.047
Combination	218.3 ± 59.2	163.8 ± 77.4	−54.5 ± 81.2	Student’s *t*-test	0.045

**Table 4:** Statistical analysis of white blood cell (WBC) counts before and after embolization.

Patient Group	Pre-SAE WBC Count (Median ± IQR)	Post-SAE WBC Count (Median ± IQR)	Shapiro–Wilk *p*-Value (Pre)	Shapiro–Wilk *p*-Value (Post)	Mann–Whitney U Test *p*-Value
Polytrauma	17.5 ± 5.2	12.5 ± 3.4	0.005	<0.001	0.777
Splenic Artery Aneurysm	9.0 ± 3.9	9.3 ± 6.7	0.039	<0.001	0.285

**Table 5:** Statistical analysis of WBC count before and after embolization by technique.

Embolization Technique	Pre-SAE WBC Count	Post-SAE WBC Count	Δ WBC Count	Test	*p*-Value
NALEA	10.1 ± 5.6	8.9 ± 2.6	−1.2 ± 4.2	Wilcoxon signed-rank	0.041
Coils	9.4 ± 5.0	8.8 ± 2.4	−0.6 ± 3.9	Student’s *t*-test	0.067
Combination	9.9 ± 5.4	8.9 ± 2.5	−1.0 ± 4.1	Wilcoxon signed-rank	0.044

## Data Availability

The raw data supporting the conclusions of this article will be made available by the authors on reques.

## Abbreviations

The following abbreviations are used in this manuscript:

AI	Artificial intelligence
CT	Computed tomography
Hb	Hemoglobin
NALEA	Non-adhesive liquid embolic agents
WBC	White blood cells
RBC	Red blood cells
SAAs	Splenic artery aneurysms
SAE	Splenic artery embolization
